# The Spanish Ion Channel Initiative (SICI) Consortium: Ten Years (2008–2018) of a Network of Excellence on Ion Channel Research

**DOI:** 10.3390/ijms19113514

**Published:** 2018-11-08

**Authors:** Antonio Felipe, Antonio Ferrer-Montiel

**Affiliations:** 1 Spanish Ion Channel Initiative (SICI), Spain; 2 Molecular Physiology Laboratory, Departament de Bioquímica i Biomedicina Molecular, Institut de Biomedicina (IBUB), Universitat de Barcelona, Avda. Diagonal 643, 08028 Barcelona, Spain; 3 Instituto de Biología Molecular y Celular (IBMC), Universitas Miguel Hernández, Av. de la Universidad s/n, 03202 Elche, Spain; 4 Instituto de Investigación, Desarrollo e Innovación en Biotecnología Sanitaria de Elche (IDiBE), Universitas Miguel Hernández, Av. de la Universidad s/n, 03202 Elche, Spain

The Spanish Ion Channel Initiative consortium (SICI, http://sici.umh.es/default.htm) started in 2008 merging the research interests of 26 Spanish multidisciplinary groups focused on ion channel investigations from the pathophysiology to drug discovery. This initiative, partially supported by public Spanish government funds, was also the promoter of the Spanish Network on Ion Channels (RECI), a multidisciplinary network that includes more than 50 Spanish groups interested on ion channels (www.reci-ionchannel.org). In the present Special Issue, some SICI members, as well as RECI colleagues, wanted to contribute with the most update knowledge of representative ion channel research in Spain for the last 10 years.

Ion channels play crucial roles in all forms of life and are highly conserved from bacteria to humans. The activity of ion channels contributes to cellular homeostasis and maintenance of health. Because of their impact on the pathophysiology of several human maladies, these proteins are the target of diverse drugs, from antiepileptic to analgesics. Furthermore, ion channel dysfunction is usually the cause of human diseases, referred to as channelopathies. More than 40 different channelopathies caused by genetic defects in ion channels have been reported thus far. Despite of the intense research in this field, there are yet a plethora of issues that remain poorly understood, such as: (i) the composition, structure and role of signaling channel complexes (channelosomes) in health and disease; (ii) the atomic structure of the pore forming subunits, of channel complexes and their dynamics; (iii) the molecular and cellular mechanisms underlying channelopathy phenotypes, as well as the implication of ion channels in the etiology of other human diseases; and, (iv) the discovery of therapeutically useful channel modulators for drug development. SICI has addressed these questions by establishing an integrative and inter-disciplinary ion channel program that brought together complementary skills. SICI pursued innovation through integration by assembling a multidisciplinary team composed of physiologists, biochemists, biophysicists, structural biologists, chemists, and pharmacologists. The major outcome of SICI has been the generation of innovative knowledge in the ion channel arena that has resulted in: (i) ground-breaking, transferable technology; (ii) original ion channel therapeutics for the treatment of human disorders; and, (iii) an integrated educational program on ion channels for graduated and postgraduate students. The integration of ion channel research via the multidisciplinary teamwork has proven to be very productive, as reflected by almost 500 scientific articles produced by the research groups that participate in SICI. In this special issue the reader may find interesting contributions from structure-function studies to cell biology, pharmacology, or physiology of ion channels.

Keeping all this in mind, we, the editors of this special issue, thank the contributors for their effort and whish that the lecture of these selected contributions will be enjoyable. These reviews and research papers bona fide reflect the outstanding level of the last 10 years research on ion channels performed by SICI and RECI members.

